# Posttraumatic Growth and Posttraumatic Depreciation: Associations with Core Beliefs and Rumination

**DOI:** 10.3390/ijerph192315938

**Published:** 2022-11-29

**Authors:** Annunziata Romeo, Lorys Castelli, Georgia Zara, Marialaura Di Tella

**Affiliations:** Department of Psychology, University of Turin, Via Verdi 10, 10124 Turin, Italy

**Keywords:** posttraumatic growth, posttraumatic depreciation, core beliefs, rumination, depression

## Abstract

Background: The positive transformation (i.e., posttraumatic growth, PTG) that can emerge after the struggles associated with a stressful life event has been widely investigated. However, less attention has been paid to the negative posttraumatic changes (i.e., posttraumatic depreciation, PTD) that might occur after a traumatic experience. This study aimed to investigate the role of a series of psychological factors (e.g., disruption of core beliefs, rumination, and depressive symptoms) in predicting PTG and PTD, separately considered. Methods: To reach this goal, 601 participants who experienced different types of traumatic events were recruited. They were asked to indicate sociodemographic and trauma-related information and to complete self-report measures assessing PTG/PTD, core beliefs, rumination, and depressive symptoms. Results: The results of regression analyses showed that gender, age, time since the trauma, core beliefs, deliberate/intrusive rumination, and depressive symptoms were significant predictors of PTG. Conversely, core beliefs, intrusive rumination, and depressive symptoms were found to be positively related to PTD. Conclusions: Taken together, these findings highlight the role that different psychological factors may play in the manifestation of the PTG and/or PTD dimensions. From a clinical perspective, professionals should pay attention to these factors when a person struggles in coping with a highly stressful experience.

## 1. Introduction

The notion that the struggle with traumatic or stressful life events can lead to significant positive transformations is widely known, and in the last 25 years a large body of research has investigated and deepened the concept of posttraumatic growth (PTG) [1,2]. It is important to specify that not all traumatic events lead to positive change. Indeed, “it is the individual’s struggle with the new reality in the aftermath of trauma that is crucial in determining the extent to which posttraumatic growth occurs” [2] (p. 5). In other words, it is not the trauma per se that is the catalyst for change, but the abrupt disintegration of one’s fundamental assumptions and the cognitive process implicated in reestablishing functional assumptions. Fundamental assumptions are a set of core beliefs that “give structure to events in an individual’s world, allow each individual to plan and predict, and contribute to how people and events in the world are perceived and understood” [3] (p. 1).

According to the theoretical model of PTG [4] and to empirical evidence [5,6,7,8,9] there is a strong positive relationship between the degree of disruption of core beliefs and the level of PTG. Effective cognitive work that considers the challenged beliefs cannot be separated from rumination. Rumination is defined as a repetitive thought, meditating on information, essentially a ‘‘chewing the cognitive cud”, and takes on a double connotation. On the one hand, intrusive rumination can be defined as a series of intrusive thoughts that are often undesired and strongly disturbing; hence, intrusive rumination implies a negative connotation and is understood as a symptom of distress [10,11]. On the other hand, repetitive thoughts can also be voluntary and controlled, and they focus on making sense of the experience. This type of rumination is defined as deliberate rumination and can be intended as a problem-solving process [10,11]. Previous studies have shown that intrusive and deliberate ruminations play different roles in influencing posttraumatic outcomes. In particular, deliberate rumination is more likely to be related to PTG, whereas intrusive rumination is more likely to be associated with posttraumatic distress (e.g., depressive symptoms or posttraumatic stress symptoms) [12,13,14].

One limitation identified in the research on posttraumatic changes is that it has focused only on the possible positive outcomes of the struggle. However, it seems easy to imagine that some individuals might also report negative posttraumatic changes as a result of their efforts to deal with stressful events. Posttraumatic depreciation (PTD) is defined as a negative change that may occur in the same domains in which people also typically report growth (e.g., relating to others, new possibilities, personal strength, spiritual change, and appreciation of life) [15]. PTG and PTD are considered two independent constructs and are both predictors of well-being [5].

While studies that investigate the disruption of core beliefs, rumination, and depressive symptoms in relation to PTG and posttraumatic stress symptoms are numerous, e.g., [9,12,13,16], less attention has been paid to the association between these variables and PTD [5,17,18].

Therefore, the main aim of the present study was to investigate the role of disruption of core beliefs, rumination (both intrusive and deliberate) about a traumatic event, and depressive symptoms both in PTG and PTD, separately considered. Specifically, our interest was to analyze if and how PTG and PTD would be predicted by those same variables. Differently from previous studies [5,17], here we used the expanded version of the Posttraumatic Growth and Depreciation Inventory (PTGDI-X) for the first time as an instrument to assess PTG and PTD in a sample of Italian adults. PTGDI-X represents the latest version of the instrument which, unlike its previous version (PTG-42) allows for a more extensive evaluation of constructs [19,20].

## 2. Materials and Methods

### 2.1. Participants and Procedure

Seven-hundred-and-fifty-nine participants were contacted and 688 completed the survey. The final sample was composed of 601 participants (response rate: 79%) who met the following inclusion criteria: being over the age of 18; being a Italian native speaker; having at least 5 years of schooling; having suffered a trauma in the past 10 years based on the DSM-5 criteria (Criterion A definition: Exposure to actual or threatened death, serious injury, or sexual violence in one (or more) of the following ways: (1). Directly experiencing the traumatic event(s); (2). Witnessing, in person, the event(s) as it occurred to others; (3). Learning that the traumatic event(s) occurred to a close family member or close friend. In cases of actual or threatened death of a family member or friend, the event(s) must have been violent or accidental; (4). Experiencing repeated or extreme exposure to aversive details of the traumatic event(s) (e.g., first responders collecting human remains, police officers repeatedly exposed to details of child abuse).

The present data were collected using an anonymous online survey from 13 March 2018 to 16 August 2019. A snowball sampling strategy was employed, wherein the participants were initially made aware of the research via online advertisements and then encouraged to disseminate the link to others. Before administering the questionnaires, the Core Beliefs Inventory and the Event-Related Rumination Inventory were translated into Italian according to the back-translation method to ensure the semantic equivalence between the Italian and English versions. Accordingly, the two measures were initially translated from English into Italian by two experts in the field who were fluent in English, and back-translated by an English university lecturer fluent in Italian. The two English versions for each measure were finally compared and differences were identified and corrected.

Afterwards, an anonymized, individual, and unique code was emailed to those who agreed to take part in the study (by providing written informed consent) to complete the online survey. Before completing the questionnaires, all participants were asked to provide demographic (i.e., age and gender) and trauma-related information (i.e., definition of a stressful event as trauma-inclusion criterion; type of traumatic event; time since the traumatic event). A list of possible traumatic experiences was included in the survey and participants were asked to choose one of them. Afterwards, the trauma definition based on DSM-5 criterion A was presented to the participants and they were asked to indicate if the selected stressful event matched the criterion. Otherwise, participants were automatically excluded from completing the survey.

The study was approved by the University of Turin Ethics Committee (Prot. n. 264810) and was conducted in accordance with the Declaration of Helsinki.

### 2.2. Measures

#### 2.2.1. Posttraumatic Growth and Depreciation

The Italian version of the PTGDI-X was employed to assess PTG and PTD dimensions [19,20]. It is a self-report instrument, consisting of 50 items. Each item is rated on a 6-point Likert scale ranging from 0 (I did not experience this change as a result of my crisis) to 5 (I experienced this change to a very great degree as a result of my crisis). The total score for each subscale ranges from 0 to 125, with higher scores indicating greater growth or depreciation. Five subscale scores can be also obtained for each of the two dimensions: Relating to Others, New Possibilities, Personal Strengths, Spiritual and Existential Change, Appreciation of Life.

The scale has shown excellent internal consistency [19] and, in our sample, the Cronbach’s alpha values were excellent for both the PTG (α = 0.93) and PTD (α = 0.93) dimensions.

#### 2.2.2. Core Beliefs

The Core Beliefs Inventory (CBI) [21] is a self-report measure, which assesses core beliefs. The CBI is composed of 9 items, each scored using a 6-point Likert-type scale ranging from 0 (not at all) to 5 (a very great degree). The total score ranges from 0 to 45, with higher scores indicating a greater disruption of core beliefs.

The CBI has shown good psychometric properties, with good internal consistency (α values = 0.82–0.87), acceptable test-retest reliability, and construct validity [21]. In our sample, Cronbach’s alpha was good for the Italian translation of the CBI (α = 0.79). 

#### 2.2.3. Intrusive and Deliberate Rumination

The Italian translation of the Event-Related Rumination Inventory (ERRI) was used to assess intrusive and deliberate rumination [11]. This is a self-report measure consisting of 40 items, which reflects two types of rumination: recent intrusive rumination (ERRI-I) and recent deliberate rumination (ERRI-D). Each item is scored using the following 4-point Likert-type scale from 1 (not at all), 2 (a little), 3 (somewhat), 4 (a lot) to 5 (extremely). Two separate total scores, ranging from 0 to 30, can be derived for intrusive and deliberate rumination. Higher scores indicate more intrusive or deliberate rumination.

The scale has shown good internal consistency, with Cronbach’s alpha coefficients ranging from 0.88 to 0.96 [11,17]. In our sample, the Cronbach’s alpha values were excellent for both the ERRI-I rumination (α = 0.97) and the ERRI-D rumination (α = 0.96).

#### 2.2.4. Depressive Symptoms

The Beck Depression Inventory-II (BDI-II) was used for the assessment of depressive symptoms [22,23]. It is a self-report measure consisting of 21 items, each scored using a 4-point Likert-type scale ranging from 0 (no symptoms) to 3 (most severe). The total ranges from 0 (no depressive symptoms) to 63 (severe depression).

The BDI-II has shown good psychometric properties, with good internal consistency (Cronbach’s alpha score = 0.91), test–retest reliability, and construct validity [24]. In our sample, Cronbach’s alpha was excellent for the Italian version of the BDI-II (α = 0.93). 

### 2.3. Statistical Analysis

Statistical analyses were conducted using the Statistical Package for Social Sciences (SPSS) version 26.0 (IBM SPSS Statistics for Windows, Armonk, NY, USA: IBM Corp.). Normal distribution was assessed using the indices of asymmetry and kurtosis. All variables resulted as normally distributed.

Descriptive data for the total sample were computed, in order to provide an overview of the sociodemographic and psychological characteristics of the participants. Descriptive data were presented as means with standard deviations for continuous variables, or frequencies with percentages for categorical variables.

In order to reach the main goal of the present study, two hierarchical multiple regression analyses were run to assess the possible significant predictors of the PTG and PTD dimensions, separately considered. PTG and PTD were used as dependent variables. The demographic variables (age and gender) theoretically expected to be associated with the PTG and PTD dimensions were included into the models to control for their possible effects [25]. The predictor groups were entered into the regression model according to the following schema: demographic and trauma-related variables (age, gender, and time since the traumatic event: first block) and psychological factors (core beliefs, intrusive and deliberate rumination, and depressive symptoms: second block). The enter method was used to include the variables of the predictor groups. Collinearity was assessed through the statistical factor of Collinearity Tolerance (CT) and Variance Inflation Factor (VIF) [26].

## 3. Results

### 3.1. Descriptive Statistics

The sociodemographic, trauma-related, and psychological data of the total sample are presented in Table 1.

The majority of the participants were women (*n* = 429; 71.4%), and the mean age of the sample was 30.91 (± 11.72). The age range of the participants was between 18 and 72 years.

With regard to the trauma characteristics, the participants reported that 39.84 (±32.77) months had passed since the traumatic event. Specifically, 37.6% of participants (*n* = 226) experienced the death of a relative or friend.

The psychological evaluation revealed that the participants reported a mean score of 59.95 (±23.72) for the growth dimension of the PTGDI-X and a mean score of 31.40 (±23.18) for the depreciation component. Furthermore, the participants reported a mean score of 27.907 (± 8.50) for the CBI and a mean score of 12.37 (±10.74) for the BDI-II. Finally, a mean score of 23.33 (±10.88) for the ERRI Intrusive scale and a mean score of 24.79 (±11.39) for the ERRI Deliberate scale were reported by the participants.

### 3.2. Multiple Regressions

To investigate the possible significant predictors of the PTG and PTD dimensions, separately considered, two hierarchical multiple regression analyses were performed. The PTG and PTD total scores were used as dependent variables in the first and second regression analyses, respectively (see Table 2).

With regard to the PTG dimension, the full model of age, gender, time since the traumatic event, core beliefs, intrusive and deliberate rumination, and depressive symptoms to predict growth (Model 2) was statistically significant: adjusted R2 = 0.3.88, F(7, 600) = 55.279, *p* < 0.001. In this case, all the included variables (age: β = 0.087, *p* = 0.009; gender: β = −0.067, *p* = 0.043; time since the traumatic event: β = 0.104, *p* = 0.029; CBI: β = 0.356, *p* < 0.001; ERRI-I: β = −0.137, *p* = 0.002; ERRI-D: β = 0.329, *p* < 0.001; BDI-II: β = −0.408, *p* < 0.001) resulted in being significant predictors of the PTG scores in the final model.

As far as the PTD dimension was concerned, the full model of age, gender, time since the traumatic event, core beliefs, intrusive and deliberate rumination, and depressive symptoms to predict depreciation (Model 2) was statistically significant: adjusted R2 = 0.443, F(7, 600) = 69.282, *p* < 0.001. Significant predictors of PTD were found to be both CBI (β = 0.071, *p* = 0.030) and ERRI-I (β = 0.204, *p* < 0.001) total scores, as well as a BDI-II total score (β = 0.574, *p* < 0.001). In all regression analyses, the statistical factor of CT and VIF showed that there were no interfering interactions between the variables.

## 4. Discussion

The present study mainly aimed to examine the role of sociodemographic factors, trauma-related information, and psychological variables (disruption of core beliefs, rumination about the event, and depressive symptoms) in PTG and PTD, separately considered. In order to achieve this goal, two hierarchical multiple regression analyses were performed. 

With regard to PTG, both being female and older, as well as having experienced the traumatic event earlier in life, were found to be associated with higher levels of growth. These findings suggest that the time elapsed since the event may favor the development of PTG and that women and older individuals seem to benefit more from a positive outcome after the trauma. 

In line with previous evidence, significant differences between men and women in terms of PTG [27] have been detected, with women reporting higher scores than men. However, in contrast with our results, previous studies have shown that younger people seem to exhibit higher levels of psychological growth compared to older individuals [7,8,28]. One explanation for these discrepant findings may be due to the fact that previous studies had often employed homogeneous samples of participants (e.g., university students) or selected a single trauma category (e.g., breast cancer or earthquake). Similarly, mixed findings were obtained with regard to the relationship between the time since the traumatic event and PTG. Indeed, several studies have shown that the time since the event was not significantly associated with PTG [28,29,30], while other studies have detected a positive association between those two constructs, e.g., [5,12,31,32]. It is possible that the relationship between PTG and time is nonlinear or that other variables account for an effect of various Time x PTG relationships. Moreover, PTG was shown to be positively associated with core beliefs and deliberate rumination, whereas it was negatively related to intrusive rumination and depressive symptoms. The importance of core beliefs disruption is well known and is still supported by recent studies, e.g., [13,33,34]. As Taku and colleagues [8] have postulated, the disruption of core beliefs plays a major role in predicting the level of PTG, suggesting that “the process of reviewing and examining core beliefs is a key catalyst for the subsequent possibility of PTG” [10] (p. 16). Significant challenge to worldview is a necessary factor, which precedes the experience of PTG. If a traumatic event does not present a challenge to previous core assumptions, then it is more unlikely that the processes necessary for growth take place [7]. 

Several studies have also suggested the significant role of rumination, intended as cognitive processing, in building the basis for PTG [5,35]. In line with our results, Freedle and colleagues [32] showed that intrusive rumination negatively predicted PTG. Conversely, other studies found that both deliberate and intrusive rumination were positive predictors of PTG [8,9,33] or that intrusive rumination, unlike deliberate rumination, was not a predictor of growth [13]. However, it seems relevant, within the scope of this paper, to discuss the different roles played by the two types of rumination based on the time since the trauma. In the time soon after the event, both intrusive and deliberate rumination seem to be relevant factors for psychological growth but only deliberate rumination is more likely to promote PTG over time. Conversely, at the present time, psychological growth seems to be positively associated with deliberate rumination and negatively associated with intrusive rumination [5,18]. In line with these assumptions, our findings have shown that individuals who intentionally activate a cognitive process, following the questioning of their basic beliefs, show greater psychological growth than those who, notwithstanding the time that has passed since the event, continue to activate involuntarily the process of intrusive rumination. 

Finally, with regard to depressive symptoms, the current study has shown that individuals currently experiencing high levels of depression report lower levels of PTG. However, as suggested by Romeo and colleagues [36], there is a nonlinear relationship between psychological distress and PTG: while depression immediately after the event appears to be a catalyst for PTG, as time passes it seems to be a hindering factor. 

Regarding PTD, the results showed that depreciation levels were positively associated with scores on core beliefs, intrusive rumination, and depressive symptoms. 

To the best of our knowledge, only three studies [5,17,18] have explored the association between PTD, core beliefs, and rumination. In line with our results, the study by Cann and colleagues [5] revealed that recent intrusive rumination was strongly related to depreciation, whereas no association was detected between PTD and deliberate rumination. Those findings suggest that depreciation could be related to a difficulty in moving from intrusive rumination to more deliberate and constructive forms of rumination. Conversely, Allbaugh and colleagues [17] found that both intrusive and deliberate rumination were significantly correlated with PTD. One possible explanation for these discrepant findings may be due to the fact that Allbaugh and colleagues [17] used a different measure to assess rumination. 

Furthermore, the study of Cann and colleagues [5] showed that the disruption of core beliefs did not contribute significantly to the prediction of PTD, suggesting that different kinds of processes may be involved in the development of growth and depreciation. Conversely, our findings show that the disruption of the assumptive world seems to be strongly correlated with both growth and depreciation. The difference between individuals who experience depreciation, rather than growth following a trauma, may be the results of the concurrent presence of high levels of depression and intrusive rumination. Partially in line with Cann and colleagues [5], we argue that PTG and PTD seem to be based on different mechanisms, except for the disruption of core beliefs, which instead appears to have an active role in the development of both posttraumatic outcomes.

The presence of depressive symptoms and intrusive rumination, together with the challenging of one’s own basic assumptions, may reflect the individual’s inability to deal constructively with the demands posed by the traumatic experience, resulting in depreciation in several existential dimensions. However, given the very few studies available, it would be useful to further investigate the processes underlying the development of PTD after a traumatic experience.

This study has some limitations that should be acknowledged. Firstly, we adopted a cross-sectional design, which does not allow certain conclusions about causal direction to be drawn. Secondly, a large proportion of the sample involved female and relatively young participants. Longitudinal studies, recruiting more heterogeneous samples, are needed to better clarify the relationship between the PTG/PTD dimensions and psychological constructs, such as core beliefs, rumination, and depressive symptoms.

Despite these limitations, these findings highlight the different role that psychological factors, such as the disruption of core beliefs, rumination (both intrusive and deliberate), and depressive symptoms, may play in the manifestation of PTG and/or PTD after a traumatic experience.

## 5. Conclusions

From a clinical perspective, clinicians should consider the degree of shattering of fundamental assumptions about life in the trauma experience. Furthermore, clinicians should also identify and support the constructive and intentional type of rumination. This may significantly enhance the process of successfully coping with highly challenging events, leading to greater psychological growth.

## Figures and Tables

**Table 1:** Sociodemographic information, trauma-related characteristics, and psychological data of the total sample (*n* = 601).

	Mean (SD)	*n* (%)	Range
**Age (years)**	30.91 (11.72)		18–72
**Gender**			
Female		429 (71.4)	
Male		172 (28.6)	
**Time since the traumatic event (months)**	39.84 (32.77)		1–120
**Types of traumatic events**			
Serious medical condition		62 (10.3)	
Being stalked		23 (3.8)	
Death of a relative/friend		226 (37.6)	
Physical or sexual assault		30 (5.0)	
Theft or mugging		10 (1.7)	
Serious illness of a relative/friend		73 (12.2)	
Natural disaster		63 (10.5)	
Being involved in a serious accident		42 (7.0)	
Others		72 (12.1)	
**Psychological evaluation**			
PTGDI-X Growth	59.95 (23.72)		0–119
PTGDI-X Depreciation	31.40 (23.18)		0–125
CBI	27.07 (8.50)		0–45
ERRI Intrusive	23.33 (10.88)		10–50
ERRI Deliberate	24.79 (11.39)		10–50
BDI	12.37 (10.74)		0–59

SD = Standard Deviation; PTGDI-X = expanded version of the Posttraumatic Growth and Depreciation Inventory; CBI = Core Beliefs Inventory; ERRI = Event-Related Rumination Inventory; BDI = Beck Depression Inventory.

**Table 2:** Hierarchical multiple regressions predicting PTG and PTD scores from sociodemographic and trauma- related variables, core beliefs, intrusive and deliberate rumination, and depressive symptoms (*n* = 601).

Predictor Variables	B	β	T	95% CI	Adj R^2^	F	ΔR^2^	ΔF
**PTG**								
**Model 1**					0.035	8.150 **	0.039	8.150 **
Age	0.161	0.080	1.972 *	0.001; 0.322				
Gender	−5.327	−0.102	−2.515 *	−9.488; −1.167				
Time since the traumatic event	0.107	0.148	3.680 **	0.050;0.164				
**Model 2**					0.388	55.279 **	0.356	87.099 **
Age	0.175	0.087	2.635 **	0.045; 0.306				
Gender	−3.521	−0.067	−2.029 *	−6.929; −0.112				
Time since the traumatic event	0.075	0.104	3.177 **	0.029; 0.122				
CBI	0.993	0.356	10.380 **	0.805; 1.181				
ERRI Intrusive	−0.299	−0.137	−3.046 **	−0.492; −0.106				
ERRI Deliberate	0.685	0.329	7.434 **	0.504; 0.866				
BDI-II	−0.901	−0.408	−11.619 **	−1.053; −0.749				
**PTD**								
**Model 1**					0.013	2.570	0.013	2.570
Age	0.050	0.025	0.619	−0.109; 0.209				
Gender	−4.291	−0.084	−2.044 *	−8.413; −0.168				
Time since the traumatic event	−0.046	−0.064	−1.580	−0.102; 0.011				
**Model 2**					0.443	69.282 **	0.437	117.807 **
Age	0.009	0.005	0.150	−0.112;0.131				
Gender	2.109	0.041	1.304	−1.067; 5.286				
Time since the traumatic event	0.032	0.045	1.439	−0.012; 0.075				
CBI	0.194	0.071	2.181 *	0.019; 0.370				
ERRI Intrusive	0.434	0.204	4.710 **	0.254; 0.613				
ERRI Deliberate	−0.43	−0.021	−0.502	−0.212; 0.126				
BDI-II	1.239	0.574	17.152 **	1.098; 1.381				

PTG = Post-Traumatic Growth; PTD = Post-Traumatic Depreciation; CBI = Core Beliefs Inventory; ERRI = Event-Related Rumination Inventory; BDI = Beck Depression Inventory. ΔR^2^ = R^2^ change; ΔF = F change; * *p* < 0.05; ** *p* < 0.01.

## Data Availability

Data will be made available on request.
